# Circulatory Syndrome: An Evolution of the Metabolic Syndrome Concept!

**DOI:** 10.2174/157340312801215773

**Published:** 2012-02

**Authors:** Ali Reza Khoshdel, Shane L Carney, Alastair Gillies

**Affiliations:** a Department of Epidemiology, Faculty of Medicine, AJA University of medical Sciences, Tehran, Iran; b John Hunter Hospital, School of Medicine and Public Health, Faculty of Health, The University of Newcastle, NSW, Australia

**Keywords:** Circulatory system, metabolic syndrome, renal function, ventricular function, arterial stiffness, anaemia.

## Abstract

The metabolic syndrome has been a useful, though controversial construct in clinical practice as well as a valuable model in order to understand the interactions of diverse cardiovascular risk factors. However the increasing importance of the circulatory system in particular the ***endothelium***, in both connecting and controlling organ function has underlined the limitations of the metabolic syndrome definition. The proposed* “Circulatory Syndrome”* is an attempt to refine the metabolic syndrome concept by the addition of recently documented markers of cardiovascular disease including renal impairment, microalbuminuria, arterial stiffness, ventricular dysfunction and anaemia to more classic factors including hypertension, dyslipidemia and abnormal glucose metabolism; all of which easily measured in clinical practice. These markers interact with each other as well as with other factors such as aging, obesity, physical inactivity, diet and smoking. The final common pathways of inflammation, oxidative stress and hypercoagulability thereby lead to endothelial damage and eventually cardiovascular disease. Nevertheless, the Circulatory (MARC) Syndrome, like its predecessor the metabolic syndrome, is only a small step toward an understanding of these complex and as yet poorly understood markers of disease.

## BACKGROUND

In medical science, a syndrome is defined as an “aggregate of symptoms and signs associated with any morbid process and constituted together they produce the picture of the disease” [1]. These components are usually caused by a unifying underlying pathology and their combination confers a risk that is different from the sum of the parts. The main purpose of such a description is to help in the diagnosis, treatment and prognosis of the disease. 

The metabolic syndrome was first described by *G.M. Reaven* in 1988 to describe a cluster of risk factors contributing to the incidence of diabetes, cardiovascular events and also mortality [2]. The definition of this syndrome remains a matter of debate and has been revised on several occasions by different organizations [3-8]. Despite such diversity, obesity, hyperglycemia, dyslipidemia and hypertension have been constant syndrome components and the central concept of such descriptions is the unity of the background pathophysiologic process and the interaction between the elements. 

### Criticisms Against Metabolic Syndrome

Several epidemiologic studies have illustrated the relationship between the metabolic syndrome, cardiovascular events and mortality [9-16]; however the syndrome was criticised by the American Diabetes Association a few years ago for its modest consistency and limited clinical application [11] and the use of the term metabolic syndrome was discouraged. Although the predictive performance of the syndrome for diabetes incidence has been stressed in several studies and meta-analyses [17], several issues remain unresolved including the presence of potential gender differences in the risk for incident diabetes associated with the metabolic syndrome and whether the metabolic syndrome offers additional prediction beyond its components [18]. Also, its value for both cardiovascular and all-cause mortality is questioned [17]. Hence, it is now important to look back at the issue to ensure about its consistency and usefulness. For instance, the following consideration must be taken for a new definition:

1. Insulin resistance has been presumed to be the common pathway for all features of the metabolic syndrome [19]; yet insulin related measurements are not standardized and vary widely [20, 21]. Despite the general belief, hyperinsulinemia and insulin resistance are not equivalent terms [11]. Besides, while 78% of individuals with metabolic syndrome have insulin resistance, only 48% of patients with insulin resistance have the metabolic syndrome [22]. Therefore the association of hyperinsulinemia and other elements of metabolic syndrome are not constant and many other factors may also play important roles as underlying mechanisms in clustering the risk factors. In other words, insulin resistance may simply be one of many abnormalities linked to a more fundamental, truly unifying pathophysiology [11].
2. Metabolic syndrome diagnosis is not always associated with higher cardiovascular risk, for example an increased risk was not observed in elderly diabetics, non-diabetic American Indians and women with suspected CV disease but normal angiography [17, 23-25]. Additionally, application of different definitions of the syndrome causes 15-20% disagreement in patients classification [11] and changes the predictive value of the syndrome diagnosis for CV disease and mortality [10, 15, 26]. Therefore the association of the included syndrome components with CV disease and with each other is uncertain. Even reports supporting the metabolic syndrome state that *“detecting the metabolic syndrome is only one part of the overall CV risk assessment and is not an adequate tool to estimate 10-year risk for coronary heart disease”*[27]. This is possibly because of many other related factors which have not been included as syndrome criteria. In fact, residual analysis of many longitudinal studies demonstrates a high unexplained variance (as much as 47%) when metabolic syndrome components were considered as independent variables [11].
3. Although several epidemiologic studies have demonstrated the relationship between metabolic syndrome and microalbuminuria, this factor was only incorporated into the World Health Organisation criteria for the syndrome. Likewise, renal failure is now recognised as an independent CV risk factor, as is anaemia, but they have not been considered as a part of the metabolic syndrome.
4. Despite a large body of evidence of the strong and independent association of endothelioarterial dysfunction with mortality, CV disease, diabetes and renal function[28-40], its impact has been overlooked in metabolic syndrome. Also, cardiac disease has been considered simply as an outcome of metabolic syndrome while it should be an interacting part of the syndrome.

By and large, the current evidence strongly suggests that the metabolic syndrome definition needs significant amendments, which should include the addition of other factors. Furthermore it must be standardized. Therefore, we introduce the term of **“*circulatory syndrome*”** instead as a more refined clinical construct since it is composed of many disease markers including cardiac, arterial and renal (respectively abbreviated as MARC) components as well as the traditional risk factors[41]. 

### Definition of the New Concept

**Fig. (1):**
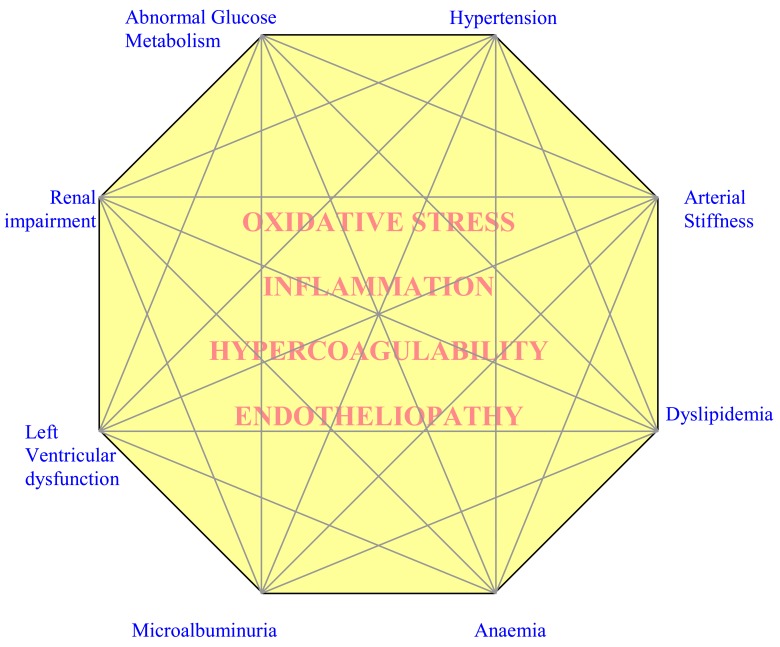
An illustrative Circulatory Syndrome; A cluster of cardiac, renal, arterial and circulatory markers of disease that are interconnected through the endothelium; the common media (underlying factors) include oxidative stress, inflammation, hypercoagulability state and endotheliopathy which contribute in the main mechanisms of the phenomena; the third dimetion (precipitating factors) include age, obesity, physical inactivity and smoking which accelerate the phenomena

Circulatory (MARC) syndrome is a cluster of *risk markers* with synergic effects. The proposed syndrome consists of eight major components (Fig. **1**):

- Abnormal glucose metabolism
- Hypertension
- Renal impairment
- Microalbuminuria
- Arterial stiffness
- Left ventricular dysfunction
- Dyslipidemia
- Anaemia

The above markers construct a network of associations while the strength of associations creates manifestation nodes. Then, the syndrome may have several facets of presentation and any given individual may exhibit some dominant features. 

The above markers can be simply and non-invasively assessed in outpatient clinical settings, although more complex assessments would be necessary for additional workups. 

All of these “markers” are expressed on a background of oxidative stress, inflammation, hypercoagulability and endotheliopathy *(underlying factors)* and can be accelerated by factors such as aging, obesity, smoking and physical inactivity *(predisposing factors)*. 

**Fig. (2):**
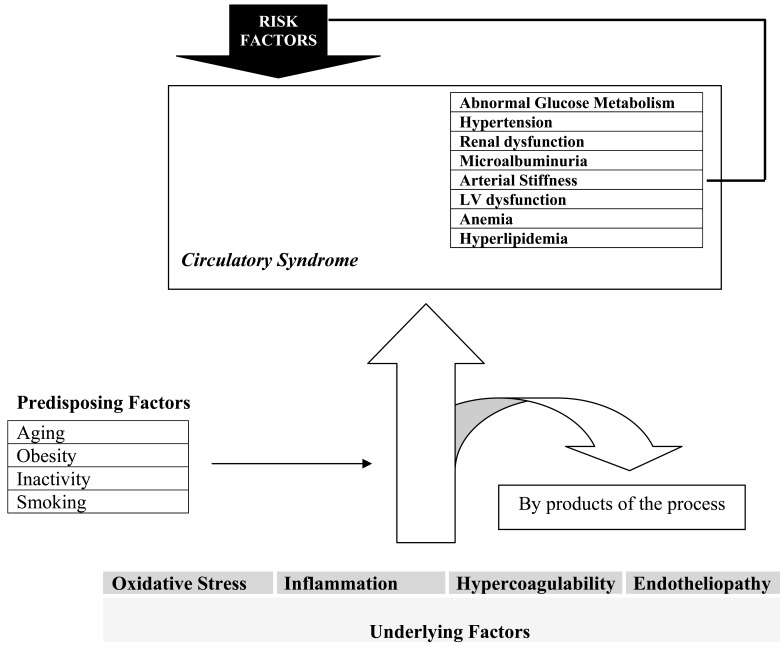
Relationships between the underlying and precipitating factors with the Circulatory Syndrome. The eight major markers of the syndrome can also play a risk factor role for other factors and progression of the syndrome.

While mechanisms underlying the circulatory (MARC) syndrome are poorly understood, it must be strongly stated that vascular-endothelial pathways link all and all are of pathological significance. Activation of the renin-angiotensin system, insulin resistance and increased sympathetic activation are all by-products of the underlying pathogenic process (Fig. **2**). Since these markers represent the extent of the underlying disease process, they could also manifest as risk factors for other components and thereby enhance their development. The final outcome in this model is the general circulatory health level of the individuals or the ability of the circulatory system to respond to body demands including exercise tolerance and ischemia related symptoms and signs. 

## RATIONALES FOR THE INCLUSION OF THE COMPONENTS

Circulatory (MARC) syndrome shares some elements with the metabolic syndrome. However it includes additional metabolic and non-metabolic factors (Table **1**).

### (1) Factors associated with abnormal glucose metabolism:

Diabetes and abnormalities in glucose metabolism are well known risk factors for cardiac, arterial and renal disease as well as anemia [42, 43]. Although insulin resistance and hyperinsulinemia can be attributed to these complications, they may occur with or without insulin resistance because several other mechanisms including advanced glycation end products, autonomic nervous instability, imbalance between the renin-angiotensin system and nitric oxide, hemodynamic changes and endothelial dysfunction with subsequent ADMA accumulation (an inhibitor for nitric oxide synthesis) and adiponectin deficiency also contribute in the process [44-46]. 

### (2) Lipid abnormalities:

Dyslipidemia including increased LDL and TG as well as low HDL is a major risk in patients with chronic renal disease, hypertension and diabetes[14, 47-49]. Genetic variants of lipoprotein lipase correlate with the presence and degree of albuminuria [50]. Dyslipidemia is an independent determinant of progression toward chronic kidney disease and is a known cardiac risk factor [51, 52]. It also contributes to arterial micro-inflammation and atherosclerosis[53]. From different perspective, the correction of lipid abnormalities can reduce albuminuria in subjects with the metabolic syndrome [54], decrease inflammatory markers[55], improve renal function[56], increase arterial compliance[57], improve left ventricular function [55] and prevent CV events[53]. It is noteworthy that obesity was not incorporated into our criteria since there is an opposite relationship between BMI and survival in CKD (reverse epidemiology) [58] and therefore less obese patients with CKD reach to ESRD.

### (3) Blood pressure abnormalities:

Hypertension is introduced as the leading risk factor of death according to WHO report of global health [59].Hypertension and altered blood pressure circadian rhythm are common co-morbidities with diabetes and pre-diabetic states as well as kidney disease[60]. BP is strongly associated with arterial stiffness and promotes left ventricular dysfunction[61] . In the setting of insulin resistance the vasodilatory effect of insulin can be lost but its renal sodium reabsorption stimulation is preserved. In addition, insulin-induced sympathetic activity increases the prevalence of hypertension in the metabolic syndrome [44]. Furthermore, while salt sensitivity is associated with impaired glucose metabolism, oxidative stress, dyslipidemia and insulin resistance [62, 63] ,it also increases efferent glomerular arteriolar tone and thereby raises glomerular capillary pressure and proteinuria [64]. Moreover, salt sensitivity induces blood pressure abnormalities via renal sodium reabsorption and sympathetic overactivity[65]. Therefore, the interlinking of salt-sensitivity and insulin resistance mechanisms with the clinical outcomes raises the possibility of another underlying mechanism joining these together.

### (4) Arterial stiffness:

Decreased arterial compliance is influenced by both atherosclerosis and arteriosclerosis, as well as functional arterial abnormalities [61, 66]. It occurs very early in the process of kidney disease and DM [67, 68], even preceding microalbuminuria [69] and has also been observed in normal individuals with a close family history of DM [68]. Recent studies have illustrated that increased central arterial stiffness in hypercholesterolemia, even in newly diagnosed individuals, is associated with low-grade systemic inflammation [70, 71]. Arterial stiffness in turn increases LV load and leads to ventricular stiffness and diastolic dysfunction [72, 73]. It has also been suggested as the linking factor between renal impairment and CV diseases [74]. Of great importance, decreased arterial compliance predicts mortality in variant patient groups, independently from other risk factors [75-78].

Furthermore, albuminuria, arterial stiffness and intima media thickness increase with the increasing number of metabolic syndrome components even before fulfilling the diagnostic criteria for the syndrome, particularly amongst subjects with type 2 DM [79]. In addition, alterations in BP circadian rhythm and BP profile including non-dipper nocturnal BP is now considered as a manifestation of arterial remodelling and is associated with other manifestation of endothelial dysfunction including mA and arterial stiffness.

### (5) Microalbuminuria

is now accepted as a marker of renal, cardiac and arterial damage being predictive for the further development of CV events, renal failure and DM [49, 74]. It is also closely associated with the prevalence of anaemia [43] , hypertension [80] and metabolic syndrome components [79]. Microalbuminuria commonly occurs early in subjects with abnormal glucose metabolism [67, 81] and is correlated with dyslipidemia [82], arterial stiffness [83, 84] and increased coagulability [85] as well as inflammatory markers [86, 87]. Furthermore the presence of microalbuminuria predicts ventricular dysfunction, coronary heart disease and exercise intolerance [88, 89]. 

### (6) Renal impairment:

Kidney function can not be isolated from the health of the heart and arteries and is also associated with the metabolic syndrome components. Alterations in glomerular structure are seen very early in the obesity-mediated metabolic syndrome[90] . Renal hemodynamic reserve is already impaired in patients with asymptomatic left ventricular dysfunction [91]. In addition, the kidney has an important role in insulin and glucose metabolism [92]and insulin resistance has a predictive value for chronic kidney disease[4, 90]. Renal function has been called the Cinderella of CV risk profile [93] and the impact of even minor renal dysfunction on CV function is now well established [74] with endothelial cell dysfunction is likely to be the linking factor between renal and cardiac disease[49, 74, 94]. However endothelial dysfunction in turn is a consequence of inflammation and oxidative stress and is accelerated by these phenomena[95] and is also correlated with a number of the metabolic syndrome components [79]. Decreased arterial compliance increases ventricular wall tension and stiffness and consequently diastolic dysfunction[73]. This in turn may lead to partial renal ischemia, followed by activation of the renin-angiotensin system and tubulointerstitial damage[94]. On the other hand, hyperfiltration which is observed in the early stages of diabetic nephropathy and hypertension [96, 97], leads to increased glomerular pressure and resultant sclerosis which in turn accelerates hypertension[60]. 

### (7) Anemia:

Anemia is a common finding in DM and has multifactorial mechanisms[43]. Early tubulointerstitial occurs which disease decreases EPO production and moreover inflammatory cytokines reduce EPO responsiveness leading to anaemia[98]. It is also associated with the level of albuminuria[43] .Anaemia in turn, increases the progression toward CKD, oxidative stress, tissue ischemia, ventricular stress and mortality[99-101]. Of interest, a recent study demonstrated the contribution of anemia to the frequent diastolic dysfunction in DM, as well as its association with brain natriuretic peptide (BNP) and suggested using this factor to identify diabetic patients at increased risk of cardiac dysfunction [99]. Therefore, accumulating evidence has introduced anemia as an important risk factor for the circulatory system. On the other hand, correction of anemia improves the prognosis in chronic kidney disease, heart failure and DM and its complications as well as decreasing mortality [101-103]

### (8) Left Ventricular dysfunction:

In contrast to the metabolic syndrome, ventricular function is proposed as an interactive part of the circulatory syndrome. This idea is supported by reports of a lack of a relationship between the metabolic syndrome and mortality in individuals who have good cardiorespiratory fitness [104]. On the other hand even a mild stage of ventricular failure, as manifested by impaired exercise response is predictive for mortality [105, 106]. Ventricular function determines blood pressure and renal perfusion and in turn is influenced by kidney function, anemia and arterial stiffness and microalbuminuria [73, 107]. Diastolic dysfunction occurs early in DM, is correlated with arterial stiffness and affects exercise response [108]. Furthermore, it has been reported that asymptomatic patients with type 2 DM have subclinical ventricular dysfunction which is related to glycated hemoglubin and LDL cholesterol [109]. Also a recent *in vitro* study demonstrated that myocyte relaxation and Ca^2+^ handling are abnormal in early uremia, leading to uremic cardiomyopathy [110].

### Additional evidence:

It is of great interest that some hypoglycaemic agents reduce blood pressure via suppression of the renin-angiotensin system and some ACE inhibitors can reduce insulin resistance in addition to reducing microalbuminuria and arterial stiffness, which raises the possibility of the presence of a common pathway for the adverse effects of hyperglycemia and hypertension[111-113]. Likewise, some lipid lowering agents may exhibit mild anticoagulant and hypotensive effects [114] and angiotensin inhibitors have anti-inflammatory actions [115] which also indicate a possible common source of these abnormalities. 

It could be expected that genetic predisposition including nephron underdosing, ACE gene polymorphism, congenital tubular defects and also some other factors such as aging, obesity and smoking produce organ damage susceptibility [48, 116-118]. 

The above evidence suggests that a genetic profile or a common pathologic process induces a network of metabolic (including alterations in glucose, salt, insulin and lipid metabolism) and hemodynamic abnormalities ( due to renin-angiotensin system stimulation, sympathetic overactivity and decreased nitric oxide bioavailability) which are followed by anaemia, hypercoagulability, tissue ischemia, arterial stiffness, hypertension, renal and cardiac dysfunction, the other features of the circulatory syndrome (Fig. **2**).

## UNDERLYING PATHOLOGY

It seems that ***inflammation*** is the fuel that “burns” the circulatory syndrome. The association between inflammatory markers and both diabetes and hypertension is so strong that these diseases has recently been redefined as inflammatory diseases, as has atheroma[119-122]. Likewise, insulin resistance has a strong link with inflammation, although additional mechanisms such as genetic factors may interfere in this relationship[123]. Additionally, inflammation is known to be a modifier of the relationship between microalbuminuria and hypertension [86, 124]. Hence, CRP has been frequently promoted as a part of the metabolic syndrome [11, 27, 125]. 

Advanced glycation end products (AGEs) which accumulate in diabetes activate inflammatory cells[119]. Moreover, inflammatory markers such as CRP are now considered to be independent predictors of diabetes [119] and its complications including left ventricular hypertrophy, endothelial dysfunction, albuminuria and renal failure [74, 95, 126, 127]. 

In addition, high LDL cholesterol induces oxidative stress and increases inflammation [128]. On the other hand HDL and Apolipoprotein A1 have anti-inflammatory and anti-oxidant properties [129]. 

There is a close relationship between inflammation and hypercoagulability [87, 130]. Furthermore, hypercoagulability is also linked to the metabolic syndrome, dyslipidemia, anaemia and even the hemodynamic response to exercise [129, 131-133]. It is associated with a poorer outcome in coronary artery disease, heart failure and is correlated with the severity of target-organ damage including renal impairment [134-136]. Moreover, diabetic and metabolic syndrome patients are at high risk for thrombotic events [137-139] and have an increased level of clotting factors including tissue plasminogen activator (tPA) and von Willenbrand Factor (vWF) and D-dimer when compared to the controls [140]. Additionally insulin and lipids may have direct effects on inhibition of coagulation and platelet function through the nitric oxide pathway, which is impaired in patients with diabetes [141].

By and large, this interlinking mesh of inflammatory mediators, oxidative stress, endotheliopathy and hypercoagulability makes a common soil for development of the circulatory syndrome.

## CLINICAL APPLICATIONS

The above description of the “circulatory (MARC) syndrome” clearly has clinical applications. Identification of commonly evaluated markers such as blood pressure, glucose and lipids in a patient should prompt a search for other circulatory syndrome markers. Suspicion about the presence of the circulatory syndrome should facilitate decision making for diagnostic procedures in asymptomatic but high risk patients. Also treatment of each syndrome component should be accompanied by management of the other components. Furthermore, any difficulty in treating one circulatory syndrome marker should probably leads to a more aggressive treatment program for other components as is currently proposed in patients with renal disease, diabetes and associated hypertension. Hence, management of circulatory syndrome would need an interdisciplinary approach with the collaboration of different medical subspecialties.

## PERSPECTIVE

Although circulatory syndrome is theoretically logical, and is supported by evidence from separate studies for each component, integrated epidemiologic studies are required to examine its consistency, validity and predictive value for adverse outcomes such as disability and mortality. Also it is important to investigate whether other markers can be added to the cluster and improve its performance. 

## CONCLUSION

Circulatory (MARC) syndrome is a cluster of interactive risk markers, combining renal, arterial, cardiac and metabolic elements into a coherent whole. These components develop on a background of inflammation, oxidative stress, hypercoagulability and endotheliopathy and while accelerated by one another, other factors including aging, obesity, physical inactivity and smoking contribute to the syndrome pathology. It’s consistency, diagnostic criteria and clinical utility still need to be investigated.

## Figures and Tables

**Table 1.** Preliminary Diagnostic Criteria for Circulatory Syndrome

**Abnormal Glucose Metabolism**
Fasting Plasma Glucose >6.1 mmol/l ; or
2hr post prandial >7.8 mmol/l

**Hypertension**
SBP≥130 mmHg; and/or
DBP≥ 85 mmHg

**GFR**
MDRD eGFR <90 ml/min/1.73 m^2^

**Microalbuminuria**
Urinary Albumin creatinine ratio (ACR) [two occasions]
>2.5 (male)
>3.5 (female)

**Arterial Stiffness**
Upper quartile for PWV, AI or ambulatory PP in the population

**Left ventricular dysfunction**
Any evidence of systolic or diastolic;
Imaging techniques or
Exercise test (MET <6, impaired systolic BP response) or
BNP> 100 pg/ml
Previous myocardial infarction

**Anemia**
Hb< 12 female
HB<13 male

**Dyslipidemia**
Triglyceride ≥ 1.7 mmol/l or
HDL<1 (male) or <1.3 (female) mmol/l or
Elevated Apolipoprotein B

GFR: Glomerular Filtration Rate, PWV: Pulse Wave Velocity, AI: Augmentation Index, PP: Pulse Pressure, SBP: Systolic Blood Pressure, DBP: Diastolic Blood Pressure, MET: Estimated multiples of resting oxygen uptake, BNP: Brain Natriuretic Peptide, HDL: High Density Lipoprotein
